# Clinical characteristics, glycemic control & quality of life of patients using Android Artificial Pancreas System (AAPS) in Brazil

**DOI:** 10.20945/2359-4292-2026-0066

**Published:** 2026-05-01

**Authors:** Gabriella Lopes Ventura, Laryssa da Silva Messias, Maria Eduarda Pereira Dantas, Milena Oliveira Leite, Débora Souto, Maria de Lourdes Passos Machado, Edson Perrotti, Thiago Mota Soares, Tassia Virginia de Carvalho Oliveira, Lenita Zajdenverg, Joana Dantas, Melanie Rodacki

**Affiliations:** 1 Universidade Federal do Rio de Janeiro, Rio de Janeiro, RJ, Brasil; 2 Universidade Tiradentes, Aracaju, SE, Brasil; 3 Departamento de Nutrologia e Diabetes, Universidade Federal do Rio de Janeiro, Rio de Janeiro, RJ, Brasil; 4 Universidade Federal de Alagoas, Maceió, AL, Brasil

**Keywords:** Type 1 diabetes, artificial pancreas, automated insulin delivery systems, quality of life, glycemic control

## Abstract

**Objective:**

This study evaluated the safety and efficacy of the Android Artificial Pancreas System (AAPS) in Brazilians with type 1 diabetes mellitus (T1D).

**Subjects and methods:**

A total of 371 participants were surveyed, including 62 AAPS users and 309 non-users. AAPS configurations included continuous glucose monitoring (CGM ), Bluetooth transmitter (MiaoMiao), and a non-automated insulin pump.

**Results:**

AAPS users had a mean Time in Range (TIR) of 78.5% ± 16.6, with HbA1c levels decreasing from 7.3% ± 1.03 to 6.5% ± 0.7 (p < 0.001). Compared to non-AAPS users, AAPS users demonstrated better glycemic control, fewer severe hypoglycemic events (p = 0.006), and improved quality of life (p < 0.0001). However, 23.08% of AAPS users had a TIR below 70%, and time in level-2 hypoglycemia exceeded recommendations.

**Conclusion:**

These findings highlight AAPS as a low-cost alternative to commercial systems, with potential to expand access to automated therapy globally, particularly in resource-limited settings.

## INTRODUCTION

Type 1 diabetes mellitus (T1D) is a chronic autoimmune disease that leads to hyperglycemia and its potential complications due to the destruction of pancreatic beta cells. Managing T1D typically involves basal-bolus insulin therapy to maintain blood glucose levels near normal and reducing the risk of complications ^(1)^. However, this treatment is complex and burdensome, requiring multiple daily insulin injections (MDI) or continuous insulin infusion systems (CIIS), frequent glucose monitoring, carbohydrate counting, and constant attention to insulin adjustments and dietary choices ^(2,3)^. Additionally, the risk of hypoglycemia can impact quality of life ^(4)^.

Automated insulin delivery systems (AID) can ease the decision-making burden for individuals with T1D, but they are expensive and often inaccessible, particularly in developing countries ^(5,6)^. These systems have been shown to improve glycemic control, increase Time in Range (TIR), reduce HbA1c levels and potentially lower the risk of diabetes-related complications ^(7-13)^. The AAPS offers a lower-cost alternative for AID, though it has not received regulatory approval in many countries ^(14,15)^. AAPS configurations include the First-generation continuous glucose monitoring (CGM), Bluetooth transmitter, and an insulin pump. It is one of the most affordable AID options and is commonly used. The First-generation CGM is an intermittent continuous (isCGM), while Bluetooth transmitter is a Bluetooth transmitter that converts the CGM into a real-time monitoring (rtCGM) system with predictive alerts, integrated with AID systems like AAPS. However, the efficacy, safety, and impact on patients’ quality of life of this AAPS configuration have not been fully evaluated in individuals with T1D.

The objective of this study was to characterize the profile of AAPS users and compare their clinical characteristics, quality of life (QoL) and glycemic control with patients with T1D using non-automated treatments in the Brazilian population.

## SUBJECTS AND METHODS

This observational study surveyed T1D patients using AAPS with First-generation CGM, Bluetooth transmitter Miaomiao), and Roche Accu-Chek Combo or Medtronic Paradigm insulin pump, recruited via social media. Participants using AAPS employed the first-generation CGM with a Bluetooth transmitter, providing continuous glucose readings during sensor wear. However, specific information regarding CGM data coverage (e.g., number of active days or hours) was not available.

A comparison group of patients not using automated insulin delivery systems was also included. Data was collected remotely through an AirTable form, covering education status, glucose control, hypoglycemia, DKA frequency, and quality of life (DQOL-Brazil). HbA1c levels reported by participants referred to values before and after AAPS use. The “before” value corresponded to the most recent HbA1c prior to initiating AAPS, and the “after” value corresponded to the most recent available measurement during system use. All HbA1c values were self-reported by participants. Data on diabetic ketoacidosis (DKA) episodes were self-reported by participants through the questionnaire. Statistical analysis was conducted using SPSS 21.0, with Mann-Whitney and Chi-square tests to compare groups, considering a significance level of <0.05. Information on whether participants received care through the public (SUS) or private health system was not collected in the questionnaire and therefore percentages for public vs. private care are not available.

This study was approved by the Research Ethics Committee of the Federal University of Rio de Janeiro (CAAE: 71962123.3.0000.5257).

## RESULTS

### Characteristics of the study group

**Figure 1:**
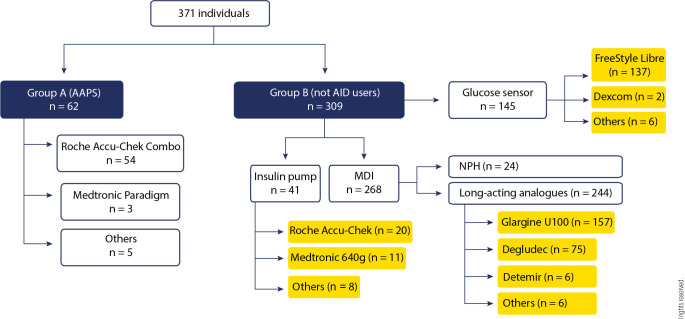
Study population and treatment characteristics of AAPS users and non-AID individuals in Brazil.

AAPS: Android Artificial Pancreas System; AID: automated insulin delivery; MDI: multiple daily injections; NPH: Neutral Protamine Hagedorn.

The study included 371 participants, divided into two groups as detailed in **Figure 1**. Among AAPS users (group A/n = 62), the mean age and disease duration were 36.4 ± 13.2 and 20.9 ± 12.1 years, respectively. In this group, 75.8% of patients aged 18 years or older and had completed higher education. For those under 18 years, 94.1% of their caregivers had completed higher education. Among AAPS users, 24% of participants had been using the system for less than 6 months, 29.6% for 6 months to 1 year, 27.7% for 1 to 2 years, and 18.5% for more than 2 years. In individuals that did not use AID (Group B/n = 309), the mean age and disease duration were 29.4 ± 11.4 years and 12.6 ± 10.1 years, respectively. Among them, 50.3% of those aged 18 years or older and 56% of the caregivers in those < 18 years old had complete higher education. In group B, 178 (57,6%) participants use glucose sensors (either flash glucose sensors or CGM). Patients’ treatments according to groups are described in **Figure 1**.

### Glycemic control

#### HbA1c levels

Among AAPS users, the mean glycated hemoglobin level (HbA1c), before starting AAPS, were 7.3% ± 1.03, which decreased to 6.5 ± 0.7% after treatment (p < 0.001).

When comparing groups, Group A demonstrated significantly lower HbA1c levels (p < 0.001), a higher proportion of individuals had HbA1c <7% (p < 0.001), and fewer severe hypoglycemia events per year (p = 0.006) than Group B. The mean weekly rates of reported hypoglycemia were similar between the groups (p = 0.73) (**Table 1**).

**Table 1:** Characteristics of the study groups

	Group A (AAPS) (n=62)	Group B (n=309)	p-value
Qol Domain Satisfaction	28 ± 11,5	41 ± 9,8	<0,0001
Qol Domain Impact	35 ± 9,3	46 ± 12,9	<0,0001
Qol Domain Social/Professional Concern	10 ± 5,8	15 ± 7,8	<0,0001
Qol Domain Concern related to T1D	8 ± 3,1	10 ± 3,8	<0,0001
A1c before AAPS	7,3% ± 1,03	7,4% ± 2,1	-
A1c after AAPS	6,5% ± 0,7	-	<0,001
A1c < 7%	75,80%	49,20%	<0,001
Hypoglycemia in a week	3,5 ± 2,2	3,3 ± 1,9	p=0,73
Number of severe hypoglycemia	0,5 ± 1,2	1,6 ± 2,1	p=0,006

QoL: quality of life. A1c: glycated hemoglobin.

#### CGM data

CGM data were collected only in group A and analyzed in 39 users. TIR, time below range (TBR, <70 mg/dL), time in level-2 hypoglycemia (<54 mg/dL), time above range (TAR, >180 mg/dL), time in level-2 hyperglycemia (>250 mg/dL), and the coefficient of variation were 77.9% ± 12.9, 4.4% ± 3.7, 1.7% ± 2.4, 17.5% ± 13.4, 4.12% ± 5.62, and 35.0 ± 7.0, respectively. Notably, 23.08% of users had a TIR below 70%, with 7.69% had a TIR below 60%, and 5.12% had a TIR below 50%.

### Treatment satisfaction and quality of life

From those using AAPS who responded, 79.2% reported being very or moderately satisfied with their current treatment, and 64.2% indicated being very or moderately satisfied with the time required for diabetes management.

When comparing quality of life (QoL), Group A had better results in all domains of the questionnaire: satisfaction (p < 0.0001), impact (p < 0.0001), social/professional concern (p < 0.0001) and concern related to T1D (p < 0.0001) (**Table 1**).

### Diabetic Ketoacidosis (DKA)

Specific data on DKA was collected from 60 patients, including 50 AAPS users (Group A) and 10 non-users (Group B). In Group A, 26% of patients experienced 1 to 2 DKA episodes per year, compared to 40% in Group B. In the group A, 74% of individuals had never had a DKA episode, versus 60% in group B. However, this data was not statistically significant (p = 0.295).

## DISCUSSION

This study assessed the safety and efficacy of a lower cost AID system in a real-world setting, with AAPS, IsCGM and Bluetooth transmitter. This analysis is particularly important, as many individuals currently use the system despite the lack of regulatory approval. If demonstrated to be safe and effective, it could provide a more affordable alternative for automated insulin delivery, particularly in developing countries where access to diabetes technology remains limited.

The findings align with previous studies that quantitatively demonstrated the impact of AAPS on glycemic control ^(15,16)^. In a study by Braune and cols. (2019), AAPS users showed an improvement in TIR, increasing from 63.8% ± 15.0 before system initiation to 79.5% ± 7.9 after its implementation. Although slightly higher than the 77.9% ± 12.9 observed in this study, this still represents significant glycemic control improvements compared to baseline levels of less than 60%. Similarly, Herzog and cols. (2020) ^(15)^ reported that do-it-yourself (DIY) closed loop users in Germany achieved a mean TIR of 79.5% ± 15.3 and reductions in HbA1c of up to 1.0%, consistent with the 0.5% HbA1c reduction observed in this study. However, in this study, the percentage of time spent in level-2 hypoglycemia (<54 mg/dL) was 1.7% ± 2.4%. While no studies have specifically analyzed this metric in AAPS users, data from commercially available artificial pancreas systems, such as the Medtronic MiniMed 780G, report significantly lower values, with TBR2 ranging from 0.5% to 0.6% ^(13)^. The slight differences in failure rates to achieve glucose control targets might be related to the various sensors used in the systems (IsCGM, Medtronic Guardian and Dexcom), the DIY nature of AAPS, combined with the absence of regulatory oversight and structured training, as well as differences in patients´ adherence and medical care. Improvements in the algorithm might also be necessary to optimize the results. Further research is needed to determine the impact of these factors on hypoglycemia rates and to improve the safety and consistency of AAPS ^(11,13,14,16,17)^.

Quality of life was assessed using the DQOL-Brazil questionnaire, showing better scores for AAPS users than non-users. Similar studies report that DIY AID systems enhance glycemic control, reduce treatment burden, and improve psychosocial outcomes, suggesting AID eases both clinical and emotional aspects of T1D management ^(15,16)^.

Better quality of life can improve adherence, reduce distress, and lower the risk of Diabetes Burnout. However, moderate satisfaction (64.2%) with the time required for diabetes management indicates the need for usability improvements. Future AAPS advancements should simplify the interface and reduce the time commitment.

Although widely used, the DQOL-Brazil primarily assesses general diabetes management and may not capture AID-specific benefits. Complementary tools like the Diabetes Distress Scale (DDS) could provide deeper insights, but they have yet to be translated into Portuguese.

Data on DKA occurrence were limited in this study, but the available data suggest that the incidence of DKA was lower among AAPS users compared to those using non-automated systems. The differences could be linked to socioeconomic and educational factors, as AAPS users in this sample had higher socioeconomic and educational levels. This may help to reduce the risk of DKA. Further studies are needed to explore this finding. It is also possible that the low frequency of reported DKA was due to heightened awareness among individuals at risk, although some AAPS users might have developed DKA due to inadequate medical supervision, as the system is DIY. It is important to note that DKA data were self-reported and not verified through medical records, which could lead to recall bias. The questionnaire collected frequency categories (e.g., 0, 1-2 per year) rather than exact counts and we do not have person-time data to calculate incidence per 100 person-years. We acknowledge that incidence rates with 95% CI would be preferable and will include them in future analyses if raw event counts and follow-up time become available.

A potential selection bias should be acknowledged, as participants were recruited through social media. This may have led to a sample composed of individuals more engaged with diabetes management and with greater access to educational and technological resources, potentially contributing to the better glycemic outcomes observed among AAPS users.

This study has limitations. Data were self-reported, and CGM data were unavailable for non-automated users. The non-automated group included individuals on various insulin regimens, introducing variability. Baseline differences between groups (e.g., age, disease duration, educational level) may have influenced the results. We understand that a multivariable analysis could be helpful, but it falls outside the descriptive scope that is the focus of this work. Furthermore, a multivariable analysis does not fit the theoretical model in which this work was conceived. Future analyses with the raw dataset are planned to perform adjusted models. We did not collect data on participants’ use of public versus private healthcare services; therefore, we could not analyze the potential influence of healthcare system type on outcomes. Additionally, factors like emotional distress, physical activity, and adherence were not assessed.

Despite this, the data remain relevant given the scarcity of AAPS research. The system is widely used globally, often without regulatory approval, leading to a DIY approach with limited medical oversight. Due to its affordability, AAPS could expand AID access, particularly in developing countries. This real-world study suggests AAPS as a viable alternative for glycemic control in T1D patients. Further randomized studies are needed to validate these findings and support regulatory approval.

In conclusion, in this observational study, individuals using AAPS with First-generation CGM, Bluetooth transmitter, and an insulin pump achieved HbA1c, TIR, TAR, and TBR levels within the targets for good diabetes control, despite a mean time in level-2 hypoglycemia slightly above target. An improvement in HbA1c was observed after initiating the system. Moreover, AAPS users with these devices demonstrated better glycemic control compared to individuals on non-automated insulin therapy, with fewer severe hypoglycemic events and better quality of life. However, some users experienced inadequate glycemic control, which may be associated with the DIY nature of the treatment. Given the significantly lower cost of AAPS compared to commercial automated insulin delivery systems, these findings suggest that this system of AAPS could serve as an alternative to expand access to automated insulin therapy globally. Proper education during system setup and medical guidance during follow-up could further reduce cases of suboptimal glycemic control.

## Data Availability

datasets related to this article will be avail-able upon request to the corresponding author.
